# Predominance of antimicrobial resistance genes and high-risk clones among Gram negatives from clinical sources in Accra-Ghana

**DOI:** 10.1371/journal.pone.0344837

**Published:** 2026-04-16

**Authors:** William Boateng, Christian Owusu-Nyantakyi, Felicia Owusu, Grebstad R. Amuasi, Quaneeta Mohktar, Pernille Nilsson, Bright Adu, Rene S. Hendriksen, Beverly Egyir

**Affiliations:** 1 Department of Bacteriology, Noguchi Memorial Institute for Medical Research, University of Ghana, Accra, Ghana; 2 Department of Immunology, Noguchi Memorial Institute for Medical Research, University of Ghana, Accra, Ghana; 3 Technical University of Denmark, National Food Institute, WHO Collaborating Centre for Antimicrobial Resistance in Foodborne Pathogens and Genomics, FAO Reference Laboratory for Antimicrobial Resistance (FAO RL), European Union Reference Laboratory for Antimicrobial Resistance (EURL-AMR), Kongens Lyngby, Denmark; Universidad San Francisco de Quito, ECUADOR

## Abstract

Gram-negative bacteria species cause increasing levels of antimicrobial resistance worldwide. Enhanced surveillance efforts are required to inform treatment decisions and monitoring of the rise and spread of antimicrobial resistant (AMR) clones, especially on the African continent, where antimicrobial resistance is known to be least tackled and controlled. In this study, whole genome sequencing was used to investigate a collection of Gram negatives recovered from clinical sources. Bacterial species were identified by Matrix-assisted Laser Desorption/Ionization Time of Flight mass spectrometry. Whole genome sequencing was performed using the Miseq illumina platform, and sequence data were analysed using free online bioinformatics tools.  Of the 182 isolates investigated, 62 resistant to at least one antibiotic were selected for whole genome sequencing. Among these, *Escherichia coli* (n=21; 33.87%) and *Klebsiella pneumoniae* (n=13; 20.97%) were the predominant Enterobacterales, while *Pseudomonas aeruginosa* (9/16; 56.25%) was most common among non-Enterobacterales. The 62 Isolates sequenced were from wound (n=37),  urine (n=19), blood (n=5), and pus (n=1). In total, 49 isolates were found to exhibit multidrug resistance (MDR). Genomic analysis revealed 126 resistance gene types, with beta-lactamase-encoding genes being the most common (56/126; 44.44%), detected in 90.32% (56/62) of organisms. *K. pneumoniae* (13/13; 100%) and *Klebsiella oxytoca* (1/1; 100%) exhibited coexisting *OqxA* and *OqxB* efflux pump genes. All *E. coli* isolates carried the MDR gene *mdf(A)*. An *Enterobacter kobei* wound isolate carried the colistin resistance gene *mcr-10*. The quaternary ammonium compound resistance gene *qacE* was present in 50% (31/62) of isolates. Additionally, 41.94% (26/62) of isolates harbored the *traT* virulence gene.. High-risk clones detected included MDR ST131 *E. coli* serotype O25:H4 (6/21; 28.57%), ST15 and ST147 *K. pneumoniae*, and ST244 *P. aeruginosa*. *Salmonella enterica* serovars Lille and Typhi recovered from blood were also identifed. The study revealed high risk clones of Gram negatives carrying multiple AMR and virulence genes. The detection of MDR pathogens and global high-risk clones, highlights the need for effective surveillance and the use of whole genome sequencing to strengthen antimicrobial resistance monitoring in our setting.

## Introduction

In recent times, numerous infections have posed challenges in treatment owing to the rise of antimicrobial resistance (AMR), a pressing global public health issue [1]. Gram-negative bacteria are medically important due to their ability to cause critical illnesses such as urinary tract infections, hospital-acquired pneumonia, bacteremia and intra-abdominal infections [2]. Of importance are the species: *Escherichia coli*, *Klebsiella pneumoniae*, *Pseudomonas aeruginosa* and *Acinetobacter baumannii* which are among the leading causes of community and hospital acquired infections [3,4]. These pathogens are largely successful because they possess mechanisms that confer resistance to multiple classes of antibiotics and an array of virulence genes [5–7]. This includes the global emergence of resistance to last-line antimicrobials such as colistin and carbapenem stemming from improper usage of the drugs. The inappropriate use of antimicrobials has resulted in escalating selective pressures in both hospital and community settings, fostering genomic evolutions in these pathogens [8].

Recent surveillance reports in Ghana have documented the rising burden of multidrug-resistant Gram-negative pathogens from clinical specimens [9]. Also, high levels of resistance to third-generation cephalosporins, aminoglycosides, and fluoroquinolones have been reported among *K. pneumoniae* and *E. coli* isolates, with Extended Spectrum Beta Lactamase (ESBL) producers becoming increasingly prevalent in bloodstream and urinary tract infections [10]. Similarly, carbapenem-resistant Acinetobacter and Pseudomonas isolates have been identified in tertiary hospitals, highlighting the emerging threat of non-Enterobacterales in critical care settings [11].

Dissemination of AMR genes occurs by vertical and horizontal transmission of which the latter involves the transfer of mobile genetic elements (MGEs) such as integrons, transposons and plasmids [12,13]. Antibiotic resistance may also occur as a result of chromosomal point mutations or protein modifications of the antibiotic targets [14]. MGEs are involved in the transfer of acquired resistance genes such as Extended Spectrum Beta Lactamase-encoding genes (*bla*_*TEM*_*, bla*_*CTX-M*_ and *bla*_*SHV*_) and carbapenemase-encoding genes (*bla*_*KPC*_*, bla*_*OXA*_*, bla*_*VIM*_*, bla*_*NDM*_) among others [12]. Gram-negative bacteria are also known to possess virulence factors such as siderophores, capsules and fimbrial adhesins, which are important for adherence, colonization and invasion of host cells during infection [15]. *LasB*, *LasA*, *PlcH*, *cnf1, FimH and traT* are examples of virulence genes involved in the establishment of critical health conditions by certain Gram-negatives [16–18].

Several Gram-negative clones with increased virulence and multidrug resistance abilities have emerged and spread over time. The pandemic *E. coli* clone ST131 has been associated with multidrug resistance and is responsible for several cases of urinary tract and bloodstream infections, among others [19]. The widespread dissemination of IncF-type plasmid carrying *E. coli* clones such as O25:H4-ST131 has contributed immensely to the success of *bla*_*CTX-M-15*_ [20]. Also, *K. pneumoniae* serotype O1 is the most common O serotype and often implicated in infections [21]. Furthermore, ST11, ST14, ST15, ST17 and ST147 are among the well characterized high risk clones in *K. pneumoniae* [22]*. P. aeruginosa* normally causes severe infections in immunocompromised patients and its serotype O6 is commonly reported [23]. The high-risk clones in *P. aeruginosa* includes ST235, ST111, ST233, ST244, ST357, ST308, ST175, ST277, ST654 and ST298. This is in accordance to their prevalence, dissemination and drug resistance profile [24]. Another noteworthy Gram-negative bacterium is *Salmonella spp*. For instance, *S. enterica* serovar Typhi-ST2, a globally recognized clone, has exhibited a gradual decrease in susceptibility to fluoroquinolones over time [25]. Also, *S. enterica* serovar Lille, originally associated with poultry, has effectively adapted to humans and has been implicated in Salmonellosis outbreaks [26,27].

Data on antimicrobial resistant bacterial species in Ghana and in Africa are mainly generated using phenotypic tools. Though this method provides general information which supports treatment decisions, it does not address the virulence and emerging resistance genes and the predominant clones of circulating pathogens in a particular setting. Whole genome sequencing (WGS) on the other hand provides comprehensive insights into the genomic makeup of AMR pathogens to effectively inform surveillance efforts. This research work utilized WGS to offer detailed information on a collection of archived Gram-negatives obtained from clinical infections, to enhance ongoing surveillance initiatives in our settings.

## Materials and methods

### Ethical consideration

Ethical approval was obtained from the Institutional Review Board of Noguchi Memorial Institute for Medical Research Review Board (University of Ghana) with approval number FW00001824. The archived isolates investigated were previously obtained and deidentified, rendering the need for informed consent not applicable to this project. The study was conducted in accordance to the regulations and principles of the committee.

### Bacteria identification, antimicrobial susceptibility testing and screening of extended spectrum beta lactamase production

A total of 182 isolates were obtained during a laboratory-based surveillance conducted between 15^th^ April 2017 and 31^st^ October 2018. The organisms were *Escherichia coli* (n = 83; 45.60%), *Klebsiella pneumoniae* (n = 30; 16.48%), *Proteus mirabilis* (n = 18; 9.89%), *Enterobacter* spp. (n = 16; 8.79%), *Salmonella* spp. (n = 8; 4.40%), *Acinetobacter* spp. (n = 4; 2.20%), *Citrobacter* spp. (n = 3; 1.65%), *Cupriavidus gilardii* (n = 1; 0.55%), *Neisseria* spp. (n = 3; 1.65%), *Pseudomonas* spp. (n = 11; 6.04%), *Providencia* spp. (n = 2; 1.10%), *Klebsiella oxytoca* (n = 1; 0.55%), *Kerstersia gyiorum* (n = 1; 0.55%) and *Alcaligenes faecalis* (n = 1; 0.55%). Only bacterial isolates that were resistant to at least one tested antibiotic, demonstrated phenotypic production of ESBLs, or were confirmed as *Pseudomonas aeruginosa* were included in the study. Isolates with no associated metadata were excluded. Based on these criteria, a total of 62 isolates were retained for further analysis. These were recovered from wound (n = 37; 59.68%), urine (n = 19; 30.65%), pus (n = 1; 1.61%), and blood (n = 5; 8.06%) samples. They originated from three hospitals (referred to as A, B, and C) located approximately 23.77 km apart in the Greater Accra Region of Ghana. Data utilized were accessed on 12^th^ March 2021 for the research. The isolates were sub-cultured on Mac-Conkey and blood agar and identified by colonial morphology, Gram staining, and confirmed by the Matrix-assisted Laser Desorption/Ionization Time of Flight mass spectrometer (MALDI-TOF MS) Biotyper™ (Bruker Daltonics, Germany). Phenotypic antimicrobial susceptibility testing was conducted using the Kirby-Bauer disk diffusion susceptibility test and results interpreted according to the Clinical and Laboratory Standards Institute (CLSI) 2018 guidelines [28]. The following antibiotic disks (Oxoid, UK) were used: ampicillin (AMP, 10 µg), tetracycline (TET, 30 µg), cefuroxime (CXM, 30 µg), cefotaxime (CTX, 30 µg), ceftriaxone (CRO, 30 µg), ceftazidime (CAZ, 30 µg), cefepime (FEP, 30 µg), cefoxitin (FOX, 30 µg), cefpodoxime (CPD, 10 µg), meropenem (MEM, 10 µg), ertapenem (ETP, 10 µg), piperacillin–tazobactam (TZP, 100/10 µg), amikacin (AK, 30 µg), gentamicin (CN, 10 µg), ciprofloxacin (CIP, 5 µg), norfloxacin (NOR, 10 µg), nalidixic acid (NAL, 30 µg), trimethoprim–sulfamethoxazole (SXT, 1.25/23.75 µg), chloramphenicol (C, 30 µg), fosfomycin (FOS, 200 µg), and nitrofurantoin (FM, 300 µg). The *K. pneumoniae* ATCC 700603 strain was used as positive control for ESBL production. The *E. coli* control strain ATCC 25922 was used as negative control. Isolates that tested positive to at least one drug in three or more drug categories were considered as multidrug resistant [29]. Isolates were screened phenotypically for production of extended spectrum beta-lactamase using the double disk diffusion test, i.e., using cefotaxime (30 µg) and ceftazidime (30 µg) alone and in combination with clavulanic acid (10 µg). An inhibition zone difference of ≥5 mm between the single and the clavulanic acid combination disks for cefotaxime and ceftazidime confirmed ESBL expression (CLSI guideline, 2018) [28].

### DNA extraction and quantification

Prior to library preparation, genomic DNA was extracted from fresh overnight bacteria cultures using the Qiagen DNA MiniAmp purification kit (Qiagen, Germany). Concentration of extracted DNA was determined by Qubit® 4.0 fluorometer using the Qubit® dsDNA High Sensitivity assay (Life Technologies, Carlsbad, US-CA) before sequencing.

### Whole-genome sequencing

Library preparation was carried out using the Illumina DNA library prep, i.e., the (M) Tagmentation Library Prep kit (Illumina Inc. San Diego, CA 92122 USA) according to the Illumina DNA library preparation reference guide (Illumina Inc., San Diego, CA, USA) to produce paired-end short-reads. Following the protocol, DNA fragmentation, tagmentation, the addition of index sequences, and amplification of indexed fragments were achieved. The assessment of library quality was performed using the 2100 bioanalyzer system (Agilent) and the concentration of libraries were determined with qPCR (Kapa Sybr Fast qPCR kit). Libraries were diluted to a concentration of 2nM, pooled, and loaded onto a 2 x 300 bp Illumina cartridge for sequencing using the MiSeq sequencing platform (Illumina, Inc., San Diego, CA, USA).

### Bioinformatics analysis

Following sequencing, the analysis of the generated FASTQ files involved trimming of indexes and reads with a quality score below 20 using Trimmomatic v.0.39 [30]. Quality control checks were performed with FastQC v.1.0 (https://www.bioinformatics.babraham.ac.uk) [31]. The trimmed raw reads were de novo assembled using Unicycler v.0.5.0 [32] and evaluated using Quast v.5.2.0 [33]. All genomes that passed the basic quality metrics of Q-score > 30, 20X minimum coverage, N50 statistic > 20000 bp, contig count < 300 and minimum contig size threshold of 200 bp were used in subsequent analysis (**S2 Table**). Following assembly, isolate identity confirmation was performed using KmerFinder v.4.1 (https://cge.food.dtu.dk/services/KmerFinder/) [34]. Antimicrobial resistance genes and chromosomal point mutations were determined using CARD v.3.0.9 (https://card.mcmaster.ca/analyze/rgi) [35] and ResFinder v.4.1 (https://cge.food.dtu.dk/services/ResFinder/) [36]. Virulence genes were determined using the Virulence Factors of Pathogenic Bacteria (VFDB) database [37] in Abricate v.0.8.10 (https://github.com/tseemann/ABRicate) and VirulenceFinder v.2.0 (https://cge.food.dtu.dk/services/VirulenceFinder/) [38]. Plasmids types were determined using PlasmidFinder v.2.1 (https://cge.food.dtu.dk/services/PlasmidFinder/) [39] and PLSDB v2021_06_23_v2 (https://ccb-microbe.cs.uni-saarland.de/plsdb/) [40]. The multi-locus sequence types (MLSTs) were predicted using MLST v.2.0 (https://cge.food.dtu.dk/services/MLST/) [41] and novel sequence-types were assigned after submission to pubMLST (https://www.PubMLST.org), EnteroBase (http://enterobase.warwick.ac.uk) [42] and Institut Pasteur’s whole-genome MLST website (http://www.pasteur.fr/mlst). Subtyping of *E. coli*, *S. enterica* and *P. aeruginosa* serotype was performed using SeroTypeFinder v.2.0 (https://cge.food.dtu.dk/services/SerotypeFinder/) [43], SeqSero v.1.2 (https://cge.food.dtu.dk/services/SeqSero/citations.php) [44] and PAst v.1.0 (https://cge.food.dtu.dk/services/PAst/) [45] respectively. The PathogenWatch database (https://pathogen.watch/) was also used to determine *Klebsiella spp.* serotypes. The phylogroups of the *E. coli* isolates were determined using the ClermonTyper web-based interface (http://clermontyping.iame-research.center/) [46]. Genome annotation of the sequences was carried out using the NCBI Prokaryotic Genome Annotation Pipeline (PGAP) (https://www.ncbi.nlm.nih.gov/genome/annotation_prok/). Default settings were employed for the tools used post-assembly unless otherwise specified. Assembled data have been deposited in the National Center for Biotechnology Information (NCBI) database under the Bioproject number PRJNA851374.

### Phylogenetic analysis

Bactinspector v.0.1.3 (https://gitlab.com/antunderwood/bactinspector) was used in the selection of the most representative reference genome for the phylogenetic analysis using the ‘closest match’ option. The reference sequence with the best hit was selected and subsequently downloaded from the NCBI database. The complete genomes with accession numbers NZ_CP010150.1 and NZ_CP009461.1 were used as reference for *E. coli* and *K. pneumoniae* phylogenetic analysis respectively. A maximum likelihood tree based on Single Nucleotide Polymorphism (SNP) of the whole-genome nucleotide sequences of *E. coli* and *K. pneumoniae* was generated using CSI Phylogeny version 1.4 [47] at default settings. The phylogenies were further visualized and annotated using iTOL v6 (https://itol.embl.de) [48].

### Statistical analysis

Microsoft Excel® (2016) was used for descriptive studies. The data generated was summarized and compared using cross-tabulation whenever applicable. Pie charts were created using the Plotly package (v.4.10.2) in R.

## Results

### Bacteria distribution, resistance and virulence gene profile, and plasmids

**Fig 1:**
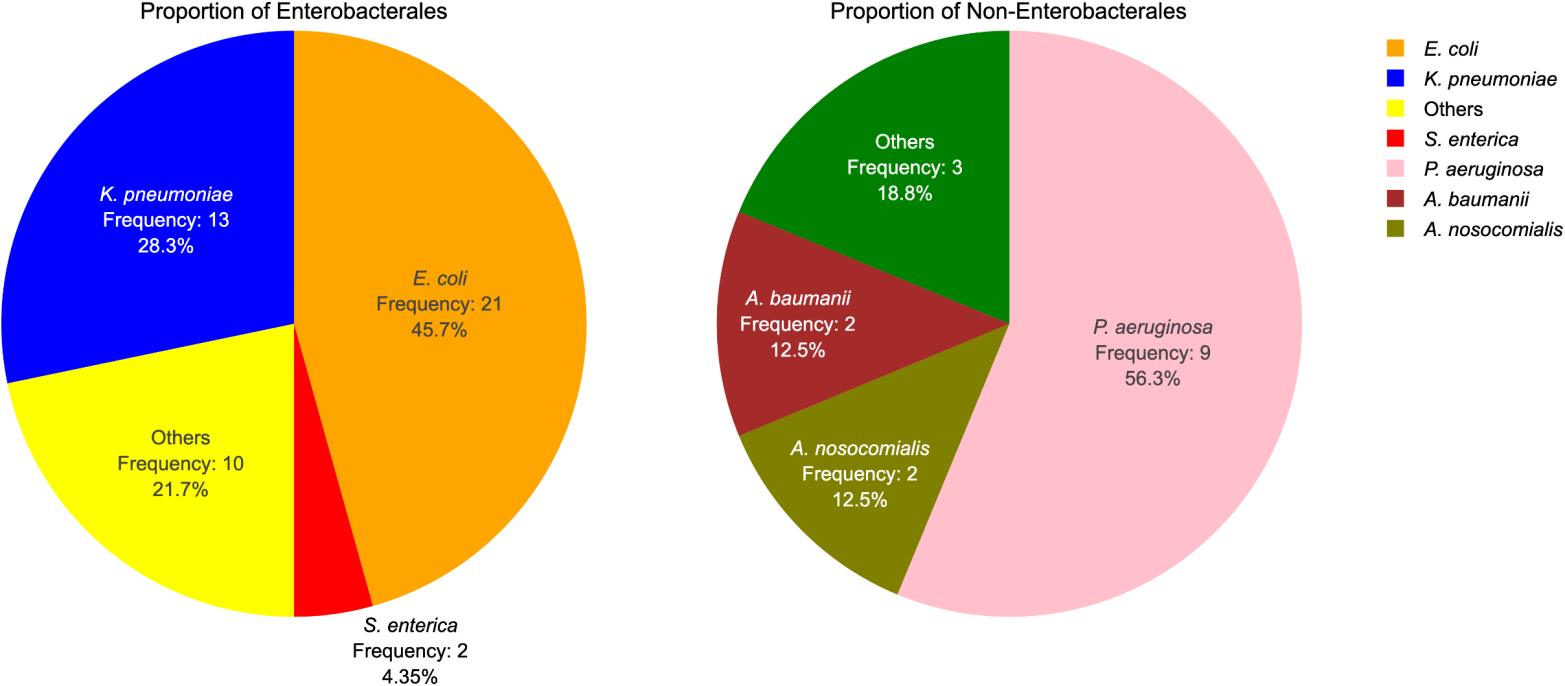
Proportion of Enterobacterales and non-Enterobacterales among the Gram-negative bacteria. Others: Enterobacterales: *P. mirabilis* (n = 2), *P. stuartii* (n = 1), *P. vermicola* (n = 1), *K. quasipneumoniae* (n = 1), *K. oxytoca* (n = 1), *Enterobacter kobei* (n = 1), *E. roggenkampii* (n = 1), *E. hormaechei* (n = 1) and *E. cloacae* (n = 1). Non-Enterobacterales: *K. gyiorum* (n = 1), *S. frequens* (n = 1) and *A. faecalis* (n = 1).

The origin and distribution of the 62 Gram-negatives investigated have been shown in **Fig 1**, **Tables 1 and 2** as well as in **S1 Table**. A total of 49 isolates exhibited multidrug resistance. Majority of the isolates (30/62; 51.61%) investigated phenotypically tested positive for ESBL production. Out of the 30 isolates that phenotypically tested positive for ESBL production, 6 did not harbor ESBL genes (**Table 3**).

**Table 1:** The genotypic characteristics of Enterobacterales isolates.

ORGANISM	ST	PLASMIDS	SEROTYPE	AMR GENES	VIRULENCE GENES
*Escherichia coli* **(n = 21)**	ST10, ST12, ST131, ST156, ST2006, ST3, ST38, ST427, ST449, ST46	nd	O15:H18, O112ac:H21, O153:H14, O9a:H23, O101:H10, O10:H28, O15:H18, O25:H4, O9:H10, O4:H1	*aac(3)-IIa, aac(3)-IId, aac(6’)-Ib-cr, aadA1, aadA2, aadA5, aph(3’)-Ia, aph(3’‘)-Ib, aph(6)-Id, bla*_*CTX-M-15*_ *, bla*_*OXA-1*_ *, bla*_*TEM-169*_ *, bla*_*TEM-1B*_ *, bla*_*TEM-1C*_ *, bla*_*TEM-33*_ *, catA1, catB3, cmlA1, dfrA1, dfrA12, dfrA17, dfrA7, dfrB4, erm(B), mdf(A), mph(A), qacE, qepA2, qepA4, sitABCD, sul1, sul2, sul3, tet(A), tet(B)*	*aar, gad, afaA, astA, fyuA, air, cea, chuA, afaD, iss, afaB, hra, cnf1, capU, aggC, lpfA, afaC, irp2, eilA, ompT, terC, iha, iucC, afaE, traT, iutA, clbB, mcbA, focC, kpsE, kpsMII_K5, sitA, senB, ireA, papA_F43, iroN, sat, papC, kpsMII_K52, usp, yfcV, kpsMII, mchB, mchC, mchF, mcmA, papA_F8, papA_F9, papA_fsiA_F16, sfaD, tcpC, vat*
*Klebsiella pneumoniae* (**n = 13**)	ST1107, ST133, ST147, ST15, ST17, ST39, ST6177, ST6178, ST6236	nd	K8:O1, K64:O2a, K19:O2afg, unk:O3b, unk:O5, unk:O1, unk:O2afg, K10:O3/O3a, K38:O3b	*aac(3)-IIa, aac(6’)-Ib3, aac(6’)-Ib-cr, aadA1, aadA16, aadA2b, aph(3’‘)-Ib, aph(6)-Id, ARR-3, bla*_*CTX-M-15*_ *, bla*_*DHA-1*_ *, bla*_*NDM-1*_ *, bla*_*OXA-1*_ *, bla*_*OXA-9*_ *, bla*_*SCO-1*_ *, bla*_*SHV-106*_ *, bla*_*SHV-11*_ *, bla*_*SHV-145*_ *, bla*_*SHV-164*_ *, bla*_*SHV-172*_ *, bla*_*SHV-179*_ *, bla*_*SHV-185*_ *, bla*_*SHV-194*_ *, bla*_*SHV-199*_ *, bla*_*SHV-26*_ *, bla*_*SHV-28*_ *, bla*_*SHV-40*_ *, bla*_*SHV-56*_ *, bla*_*SHV-59*_ *, bla*_*SHV-67*_ *, bla*_*SHV-75*_ *, bla*_*SHV-78*_ *, bla*_*SHV-79*_ *, bla*_*SHV-85*_ *, bla*_*SHV-89*_ *, bla*_*SHV-94*_ *, bla*_*SHV-96*_ *, bla*_*SHV-98*_ *, bla*_*TEM-104*_ *, bla*_*TEM-1A*_ *, bla*_*TEM-1B*_ *, catA1, catA2, catB3, dfrA14, dfrA15, dfrA16, dfrA27, erm(B), fosA, fosA5, mph(A), OqxA, OqxB, qacE, qnrB1, qnrB2, qnrB4, qnrS1, sul1, sul2, tet(A), tet(D)*	*iutA, fyuA, traT, irp2*
*Salmonella enterica* **(n = 2)**	ST9685, ST2	IncFIA(HI1), IncHI1A, IncHI1B(R27)	Lille (7:z38), Typhi (9:d)	*aac(6’)-Iaa, bla*_*TEM-1B*_ *, catA1, dfrA15, qacE, sul1, tet(B), fosA7*	sspH2, sopD2, fimI, fimC, fimD, fimH, fimF, ssaQ, ssaR, ssaS, ssaT, ssaU, cdtB, tviB, tviC, tviD, tviE *
*Proteus mirabilis* **(n = 2)**	n.s.a	nd	nd	*aadA2b, aph(3’‘)-Ib, aph(6)-Id, cat, dfrA32, ere(A), floR, sul2, tet(C), tet(J), aadA1, bla*_*CARB-2*_ *, cat, dfrA1, dfrA15,*	nd
*Enterobacter cloacae* **(n = 1)**	ST1	IncFIA(HI1)	nd	*aadA1, aph(3’‘)-Ib, aph(6)-Id, bla*_*CMH-3*_ *, bla*_*DHA-1*_ *, dfrA15, fosA, qacE, qnrB4, sul1*	nd
*Enterobacter hormaechei* **(n = 1)**	ST2157	nd	nd	*bla*_*ACT-7*_ *, fosA*	*iroN*
*Enterobacter roggenkampii* **(n = 1)**	ST131	IncFIB(K), IncY	nd	*aadA2, bla*_*DHA-1*_ *, bla*_*MIR-1*_ *, catA1, dfrA12, fosA, mph(A), qacE, qnrB4, sul1, tet(A)*	nd
*Enterobacter kobei* **(n = 1)**	ST56	Col(pHAD28), IncFIB(K), IncFII(Yp)	nd	*bla*_*ACT-9*_ *, fosA, mcr-10*	nd
*Klebsiella oxytoca* **(n = 1)**	ST419	IncFIB(pHCM2)	nd	*bla*_*OXY-2–6*_	nd
*Klebsiella quasipneumoniae* **(n = 1)**	ST841	Col(pHAD28), Col440I, IncFIB(K), IncR	nd	*OqxA, OqxB, bla*_*OKP-B-15*_ *, bla*_*OKP-B-2*_ *, catA1, fosA, tet(A)*	*iutA*
*Providencia stuartii* **(n = 1)**	n.s.a	Col3M	nd	*aac(2’)-Ia, aadA1, aph(3’)-Ia, catA3, dfrA1, qnrD1, sul3, tet(B)*	nd
*Providencia vermicola* **(n = 1)**	n.s.a	nd	nd	*ARR-2, aac(6’)-Ib-cr, aadA1, aph(3’)-VI, bla*_*DHA-1*_ *, bla*_*NDM-1*_ *, bla*_*OXA-10*_ *, dfrA14, qacE, qnrA1, rmtC, sul1*	nd

**Definitions: nd**: not determined; **n.s.a**: no scheme available; **unk**: unknown; *: among others (remaining genes in **S1 Table**). The Enterobacterales isolates were recovered from the following clinical specimens: *E. cloacae* (urine, n = 1), *E. hormaechei* (urine, n = 1), *E. kobei* (wound, n = 1), *E. roggenkampii* (wound, n = 1), *E. coli* (pus, n = 1; urine, n = 3; wound, n = 17), *K. oxytoca* (wound, n = 1), *K. pneumoniae* (blood, n = 1; urine, n = 9; wound, n = 3), *K. quasipneumoniae* (urine, n = 1), *P. mirabilis* (wound, n = 2), *P. stuartii* (wound, n = 1), *P. vermicola* (wound, n = 1), and *S. enterica* (pus, n = 2)*.*

**Table 2:** The genotypic characteristics of non-Enterobacterales isolates.

ORGANISM	ST	PLASMIDS	SEROTYPES	AMR GENES	VIRULENCE GENES
*Pseudomonas aeruginosa* **(n = 9)**	ST4333, ST3662, ST244, ST4334, ST4332	pHOU1−1	O7, O11, O5, O6	*aph(3’)-IIb, bla*_*OXA-114c*_ *, bla*_*OXA-50*_ *, bla*_*OXA-395*_ *, bla*_*OXA-396*_ *, bla*_*OXA-114g*_ *, bla*_*OXA-485*_ *, bla*_*PAO*_ *, bla*_*OXA-494*_ *, bla*_*OXA-488*_ *, catB7, fosA, crpP*	lasA, tse1, pscL, pscK, pscJ, pscI, pscH, pscG, pscF, pscE, pscD, pscC, pscB, exsD, exsA, exsB, exsE, exsC, popD, popB, pcrH, pcrV, pcrG, pcrR, pcrD, pcr4, pcr3, pcr2, pcr1, flhF, flhA, flhB, fliR, fliQ *
*Acinetobacter baumannii* **(n = 2)**	ST2213	nd	nd	*bla*_*ADC-25*_ *, bla*_*OXA-70*_	nd
*Acinetobacter nosocomialis* **(n = 2)**	ST782, ST279	pVB11737_6, pWP2-W18-ESBL-11_3, pVB11737_5	nd	*bla*_*ADC-25*_ *, tet(39), sul2*	*nd*
*Alcaligenes faecalis* **(n = 1)**	n.s.a	pESBL176, pESBL96	nd	*aac(6’)-Ib-cr, aac(6’)-Ib3, aadA1, ant(2’‘)-Ia, aph(3’‘)-Ib, aph(6)-Id, bla*_*CARB-4*_ *, catB3, dfrA15, dfrA17, floR, qacE, sul1, tet(A), tet(G)*	nd
*Kerstersia gyiorum* **(n = 1)**	n.s.a	nd	nd	*catB3*	nd
*Stutzerimonas frequens* **(n = 1)**	n.s.a	nd	nd	nd	*algA, algU, flgG, flgI, algB, xcpT, xcpS, xcpR, waaF, pilJ, pilH, pilG, pilU, pilT, pilR, algR, algC, fliE, fliG, fliI, fliM, fliN, fliP, fliQ, flhA, fleN, fliA, motC, fleQ*

**Definitions: nd**: not determined; **n.s.a**: no scheme available; **unk**: unknown; *: among others (remaining genes in **S1 Table**). The non-Enterobacterales isolates were recovered from various clinical specimens: *A. baumannii* (blood, n = 1; urine, n = 1), *A. nosocomialis* (urine, n = 2), *A. faecalis* (wound, n = 1), *K. gyiorum* (wound, n = 1), *P. aeruginosa* (wound, n = 9), and *S. frequens* (blood, n = 1).

**Table 3:** The antibiotype and genotypic resistance profile of phenotypically confirmed ESBL-positive isolates.

ID	Organism	Antibiotype	Antibiotic Resistance Genes
*Isolate_1	ECL	AMP-CXM-CTX-NAL-CAZ-CPD-ETP	*aadA1, aph(3’‘)-Ib, aph(6)-Id, bla*_*CMH-3*_ *, bla*_*DHA-1*_ *, dfrA15, fosA, qacE, qnrB4, sul1*
*Isolate_2	EH	CRO-AMP-CXM-CTX-FM-NAL-CPD	*bla*_*ACT-7*_ *, fosA*
Isolate_7	KP	CRO-AMP-CXM-CTX-CAZ-CPD-ETP	*OqxA, OqxB, bla*_*SHV-185*_ *, fosA*
Isolate_9	KP	CRO-AMP-CXM-CTX-NOR-FOS-FM-NAL-CAZ-CPD	*OqxA, OqxB, aac(3)-IIa, aadA2b, bla*_*CTX-M-15*_ *, bla*_*SHV-172*_ *, bla*_*SHV-94*_ *, bla*_*SHV-96*_ *, bla*_*TEM-1B*_ *, catA2, dfrA14, dfrA16, fosA, qacE, qnrS1, sul1, sul2, tet(D)*
Isolate_12	KP	CRO-AMP-CXM-CTX-FM-NAL-CAZ-CPD	*ARR-3, OqxA, OqxB, aac(3)-IIa, aac(6’)-Ib-cr, aadA16, aph(3’‘)-Ib, aph(6)-Id, bla*_*CTX-M-15*_ *, bla*_*SHV-40*_ *, bla*_*SHV-56*_ *, bla*_*SHV-79*_ *, bla*_*SHV-85*_ *, bla*_*SHV-89*_ *, bla*_*TEM-1B*_ *, catA2, dfrA27, erm(B), fosA, mph(A), qacE, qnrB2, sul1, sul2, tet(D)*
Isolate_13	KP	CRO-AMP-CXM-CTX-NOR-FM-NAL-CAZ-CPD	*OqxA, OqxB, aac(3)-IIa, aac(6’)-Ib-cr, aadA1, bla*_*CTX-M-15*_ *, bla*_*OXA-1*_ *, bla*_*SHV-11*_ *, bla*_*SHV-67*_ *, bla*_*TEM-104*_ *, bla*_*TEM-1B*_ *, catA1, catB3, dfrA15, erm(B), fosA, mph(A), qacE, sul1, sul2, tet(D)*
Isolate_15	KP	CRO-AMP-CXM-CTX-FOS-FM-NAL-CPD-ETP	*OqxA, OqxB, bla*_*SHV-185*_ *, fosA*
Isolate_16	KP	CRO-AMP-CXM-CTX-FM-CAZ-CPD	*OqxA, OqxB, aac(3)-IIa, aadA1, aph(3’‘)-Ib, aph(6)-Id, bla*_*CTX-M-15*_ *, bla*_*SCO-1*_ *, bla*_*SHV-75*_ *, bla*_*TEM-1B*_ *, catA2, dfrA14, fosA, qacE, qnrB1, sul1, sul2, tet(D)*
*Isolate_22	AB	CRO-AMP-CXM-CTX-FOS-FM-CPD-ETP	*bla*_*ADC-25*_ *, bla*_*OXA-70*_
*Isolate_23	AN	CRO-AMP-CXM-CTX-FOS-FM-CPD-ETP	*bla*_*ADC-25*_ *, tet(39)*
Isolate_25	EC	AMP-TET-CXM-CIP-C-NOR-SXT-ETP-CAZ	*aadA1, aadA2, bla*_*CTX-M-15*_ *, catA1, cmlA1, dfrA12, erm(B), mdf(A), mph(A), qacE, sul1, sul3, tet(B)*
Isolate_28	EC	AMP-TET-CXM-CTX-CIP-C-NOR-SXT-ETP-CAZ	*bla*_*CTX-M-15*_ *, bla*_*TEM-1B*_ *, catA1, dfrB4, mdf(A), qacE, qepA2, sul1, tet(B)*
Isolate_29	EC	TET-CXM-CTX-CIP-C-NOR-SXT-ETP-CAZ	*bla*_*CTX-M-15*_ *, bla*_*TEM-1B*_ *, catA1, dfrB4, mdf(A), qacE, qepA2, sul1, tet(B)*
Isolate_33	EC	AMP-TET-CXM-CTX-CIP-C-NOR-SXT-ETP-CAZ	*aac(6’)-Ib-cr, aadA5, bla*_*CTX-M-15*_ *, bla*_*OXA-1*_ *, catB3, dfrA17, erm(B), mdf(A), mph(A), qacE, sul1, tet(B)*
Isolate_34	KP	AMP-TET-CXM-CTX-MEM-CIP-C-NOR-SXT-CN-CAZ	*OqxA, OqxB, aac(3)-IIa, aac(6’)-Ib-cr, aph(3’‘)-Ib, aph(6)-Id, bla*_*CTX-M-15*_ *, bla*_*OXA-1*_ *, bla*_*SHV-106*_ *, bla*_*SHV-28*_ *, bla*_*TEM-1B*_ *, catB3, fosA, qnrB1, sul2, tet(A)*
Isolate_36	EC	AMP-TET-CXM-CTX-CIP-C-NOR-SXT-ETP-CAZ	*aadA1, aadA2, bla*_*CTX-M-15*_ *, catA1, cmlA1, dfrA12, erm(B), mdf(A), mph(A), qacE, sul1, sul3, tet(B)*
*Isolate_38	PA	AMP-TET-CXM-CTX-MEM-AK-CIP-C-NOR-CN-ETP-FOX-CAZ	*bla*_*OXA-114c*_ *, bla*_*OXA-114g*_
Isolate_39	EC	AMP-TET-CXM-CTX-MEM-CIP-C-NOR-SXT-ETP-CAZ	*aadA1, aadA2, bla*_*CTX-M-15*_ *, catA1, cmlA1, dfrA12, erm(B), mdf(A), mph(A), qacE, sul1, sul3, tet(B)*
Isolate_40	EC	AMP-TET-CXM-CTX-MEM-CIP-C-NOR-SXT-ETP-CAZ	*aadA1, aadA2, bla*_*CTX-M-15*_ *, catA1, cmlA1, dfrA12, erm(B), mdf(A), mph(A), qacE, sul1, sul3, tet(B)*
Isolate_41	EC	AMP-TET-CIP-C-NOR-SXT-CN-ETP	*aac(3)-IId, aadA5, aph(3’‘)-Ib, aph(3’)-Ia, aph(6)-Id, bla*_*TEM-1B*_ *, catA1, dfrA17, mdf(A), mph(A), qacE, sitABCD, sul1, sul2, tet(B)*
Isolate_44	EC	AMP-TET-CXM-CTX-AK-CIP-NOR-SXT-CN-ETP-CAZ	*aac(3)-IIa, aac(6’)-Ib-cr, aadA5, bla*_*CTX-M-15*_ *, bla*_*OXA-1*_ *, catB3, dfrA17, mdf(A), mph(A), qacE, sitABCD, sul1, tet(A)*
Isolate_45	EC	AMP-TET-CXM-CTX-AK-CIP-NOR-SXT-CN-ETP-CAZ	*aac(3)-IIa, aac(6’)-Ib-cr, aadA5, bla*_*CTX-M-15*_ *, bla*_*OXA-1*_ *, catB3, dfrA17, mdf(A), mph(A), qacE, sitABCD, sul1, tet(A)*
Isolate_47	EC	AMP-TET-CXM-CTX-AK-CIP-C-NOR-SXT-CN-ETP-CAZ	*aac(3)-IIa, bla*_*CTX-M-15*_ *, catA1, mdf(A), sitABCD, tet(B)*
Isolate_53	EC	AMP-TET-CXM-CTX-CIP-NOR-SXT-CN-ETP-CAZ	*aac(3)-IIa, aac(6’)-Ib-cr, aadA5, bla*_*CTX-M-15*_ *, bla*_*OXA-1*_ *, catB3, dfrA17, mdf(A), mph(A), qacE, sitABCD, sul1, tet(A)*
Isolate_55	EC	AMP-TET-CXM-CTX-MEM-CIP-C-NOR-SXT-ETP-CAZ	*aadA1, aadA2, bla*_*CTX-M-15*_ *, catA1, cmlA1, dfrA12, erm(B), mdf(A), mph(A), qacE, qepA4, sul1, sul3, tet(B)*
*Isolate_57	PA	AMP-TET-CXM-CTX-CIP-C-NOR-SXT-CN-ETP-CAZ	*aac(6’)-Ib-cr, aac(6’)-Ib3, aadA1, ant(2’‘)-Ia, aph(3’‘)-Ib, aph(6)-Id, bla*_*CARB-4*_ *, catB3, dfrA15, dfrA17, floR, qacE, sul1, tet(A), tet(G)*
Isolate_58	KP	AMP-TET-CXM-CTX-MEM-CIP-NOR-SXT-CN-ETP-CAZ	*OqxA, OqxB, aac(3)-IIa, aac(6’)-Ib-cr, aph(3’‘)-Ib, aph(6)-Id, bla*_*CTX-M-15*_ *, bla*_*OXA-1*_ *, bla*_*SHV-106*_ *, bla*_*SHV-28*_ *, bla*_*TEM-1B*_ *, catB3, fosA, qnrB1, sul2, tet(A)*
Isolate_59	EC	AMP-TET-CXM-CTX-MEM-CIP-NOR-SXT-CN-ETP-CAZ	*aac(3)-IIa, aac(6’)-Ib-cr, aadA5, bla*_*CTX-M-15*_ *, bla*_*OXA-1*_ *, catB3, dfrA17, mdf(A), mph(A), qacE, sitABCD, sul1, tet(A)*
Isolate_60	EC	AMP-TET-CXM-CTX-CIP-C-NOR-SXT-ETP-CAZ	*aadA1, aadA2, bla*_*CTX-M-15*_ *, catA1, cmlA1, dfrA12, erm(B), mdf(A), mph(A), qacE, sul1, sul3, tet(B)*
Isolate_61	EC	AMP-TET-CXM-CTX-CIP-C-NOR-SXT-CN-ETP-CAZ	*aac(3)-IIa, aac(6’)-Ib-cr, aadA5, bla*_*CTX-M-15*_ *, bla*_*OXA-1*_ *, catB3, dfrA17, mdf(A), mph(A), qacE, sitABCD, sul1, tet(A)*

**Abbreviations**: NAL- Nalidixic acid; CPD- Cefpodoxime; ETP- Ertapenem; CRO- Ceftriaxone; FM- Nitrofurantoin; NOR- Norfloxacin; FOS- Fosfomycin; AK- Amikacin; CIP- Ciprofloxacin; CAZ- Ceftazidime; CTX- Cefotaxime; TE- Tetracycline; CXM- Cefuroxime; CN- Gentamicin; MEM- Meropenem; FOX- Cefoxitin; SXT- Sulfamethoxazole-trimethoprim; C- Chloramphenicol; AMP-Ampicillin; *- Isolate not harboring an ESBL gene; ECL- *Enterobacter cloacae*; EH- *Enterobacter hormaechei*; KP- *Klebsiella pneumoniae*; AN- *Acinetobacter nosocomialis*; EC- *Escherichia coli*; PA- *Pseudomonas aeruginosa.*

A total of 126 resistance gene types were identified among the isolates including beta-lactamase encoding genes (56/126; 44.44%). The ESBL gene, *bla*_*CTX-M-15*_ (22/62; 35.48%) was common. The *bla*_*NDM-1*_ carbapenemase gene was found in *Klebsiella pneumoniae* (1/13; 7.69%) and *Providencia vermicola* (1/1; 100.00%). Other resistant genes detected include *bla*_*OXA-1*_ (12/62; 19.35%), *bla*_*TEM-1B*_ (11/62; 17.74%), *sul1* (30/62; 48.39%) and *fosA* (25/62; 40.32%)*.* The most predominant aminoglycoside resistance gene was *aadA1* (17/62; 27.42%). The colistin resistance gene *mcr-10* was found in *Enterobacter kobei*. Efflux pump associated genes *OqxA* and *OqxB,* were found to co-exist in *K. pneumoniae* (13/13; 100.00%) and *Klebsiella oxytoca* (1/1; 100.00%). *mdf (A)*, a multi-drug resistance gene was detected in all *E. coli* isolates (**Table 1** and **S1 Table**). The *qacE* gene that confers resistance to quaternary ammonium compounds (disinfectants) was found in half (31/62; 50.00%) of the isolates.

Twenty-six isolates possessed *traT* (n = 26; 41.94%) virulence gene. An *S. enterica* isolate was observed to possess the highest number of virulence genes (**S1 Table**).

IncFIB (AP001918) (17/46; 36.96%) was the most prevalent plasmid replicon among the Enterobacterales followed by IncFIA (16/36; 44.44%). Interestingly, both were found only in *E. coli. P. aeruginosa* recorded a high number of plasmids with pHOU1−1 being the most common (**S1 Table**).

### Serotype, multi-locus sequence type and phylogroup

**Fig 2:**
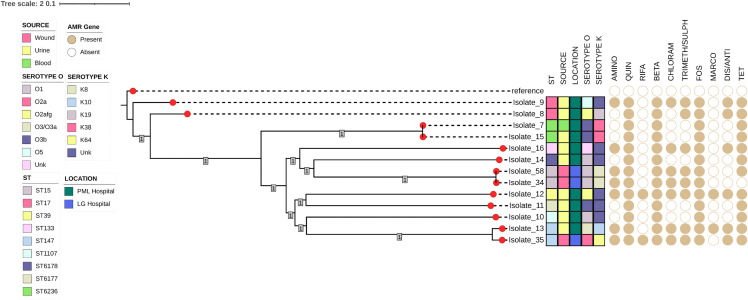
Single Nucleotide Polymorphism (SNP) based phylogenetic tree showing the evolutional relationship among 13 *K. pneumoniae* isolates. The AMR drug classes included in the visualization are aminoglycoside (AMINO), quinolone (QUIN), rifamycin (RIFA), beta-lactam (BETA), chloramphenicol (CHLORAM), trimethoprim/sulphonamide (TRIMETH/SULPH), fosfomycin (FOS), macrolide (MACRO), disinfectant/antiseptic (DIS/ANTI) and tetracycline (TET). Bootstrap values ranged from 0 to 1, with all branches showing strong support (value = 1).

Klebsiella capsular serotyping identified 5 serotypes (K8, K38, K10, K19 and K64) with K38 (2/13 15.38%) and K8 (2/13; 15.38%) having the highest frequencies. Among the 13 *K. pneumoniae* isolates, lipopolysaccharide serotypes O1, O2a, O2afg, O3/O3a, O3b and O5 were detected with O1 (4/13; 30.77%) as the common serotype. The K38: O3b (2/13; 15.38%) and K8: O1 (2/13; 15.38%) serotypes occurred frequently among the *K. pneumoniae* strains (**Fig 2**, **Table 1** and **S1 Table**). Among the *E. coli* strains*,* H23 (6/21; 28.57%) and H4 (6/21; 28.57%) were the most predominant flagellar serotypes (H1, H23, H10, H4, H14, H18, H21and H28)*.* Furthermore, *E. coli* strains O25: H4 (n = 6; 28.57%) and O9a: H23 (n = 6; 28.57%) were found to be predominant. *S. enterica* serotypes Typhi (9: d) [1/2; 50.00%] and Lille (7: z38) [1/2; 50.00%] were also detected. Serotype O6 *P. aeruginosa* was common among the *P. aeruginosa* isolates (**Table 2** and **S1 Table**).

*E. coli* ST131 (6/21; 28.57%) and ST2006 (6/21; 28.57%) were the dominant MLSTs followed by ST156 (2/21; 2.56%). ST147, ST15, ST17 and ST6236 had the highest frequencies among *K. pneumoniae* each with a proportion of 15.38% (2/13). ST2 was identified among *Salmonella enterica* isolates. *P. aeruginosa* strains belonging to ST244 (2/9; 22.22%) and ST3662 (2/9; 22.22%) were also identified. Novel sequence types were identified for *K. pneumoniae* [ST6178 (n = 1) and ST6236 (n = 2)], *A. baumanii* [ST2213 (n = 2)], *E. hormaechei* [ST2157 (n = 1)] and *P. aeruginosa* [ST4332 (n = 2), ST4333 (n = 1) and ST4334 (n = 2)]. However, MLST schemes for *Alcaligenes faecalis*, *Kerstersia gyiorum*, *Providencia stuartii*, *Providencia vermicola*, *Stutzerimonas frequens* and *Proteus mirabilis* were not available.

**Fig 3:**
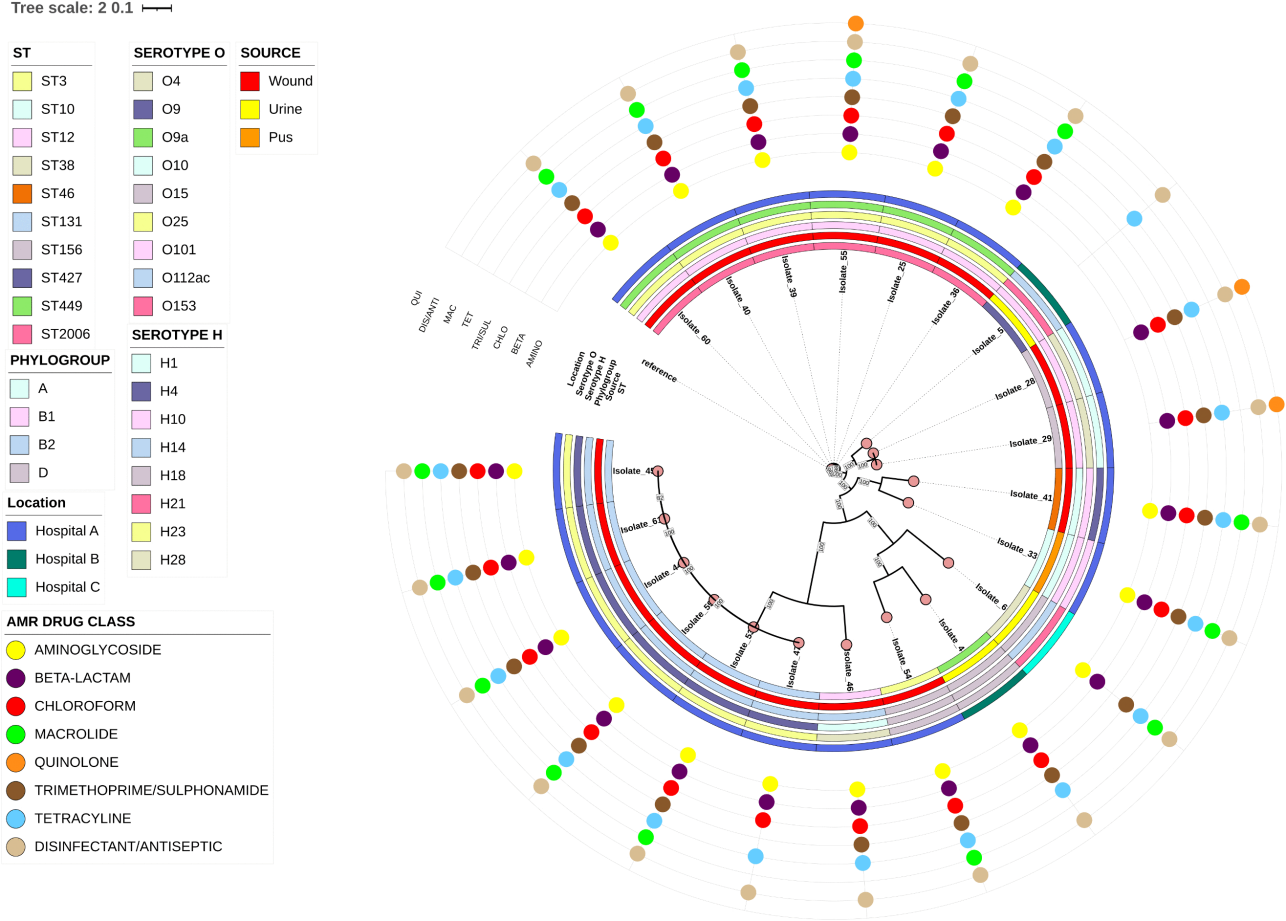
A maximum likelihood tree based on the Single Nucleotide Polymorphism (SNP) analysis of 21 *E. coli* isolates. The figure was visualized and annotated using iTOL v6. The annotated rings show characteristics of the isolates: Sequence type, Source, Phylogroup, Serotype H, Serotype O and AMR drug classes (outer rings). On the outer rings, the AMR drug classes are as follows: Quinolone (QUI), Disinfectant/Antiseptic (DIS/ANTI), Macrolide (MAC), Tetracycline (TET), Trimethoprim/Sulphonamide (TRI/SUL), Chloramphenicol (CHLO), Beta Lactam (BETA) and Aminoglycoside (AMINO). All branches were well supported with a minimum bootstrap value of 80 on a scale of 0 to 100.

Phylogroups of *E. coli* was determined in-silico using the Clermont phylogenetic typing scheme. The proportions of phylogroups identified were: B1 (9/21; 42.86%), B2 (7/21; 33.33%), D (3/21; 14.29%) and A (2/21; 9.52%) (**Fig 3**).

### Phylogenetic analysis

All the branches of the *E. coli* and *K. pneumoniae* evolutionary trees were well supported with a bootstrap value above 80 (or 0.8). Phylogenetic analysis of the *K. pneumoniae* isolates revealed that the pairwise SNP distances among the isolates ranged from 1 to 20767. Some isolates from the same location showed very close genetic relatedness of less than 10 SNPs. The pairwise SNP distance of Isolate_58 and Isolate_34 from Hospital A was four (4), and the isolates were recovered from different sources (blood and urine). Both isolates also exhibited comparable resistance genes, encompassing those responsible for resistance to aminoglycosides, quinolones, beta-lactams, chloramphenicols, sulphonamides, fosfomycins, and tetracyclines. These isolates clustered together on the tree. Isolate_15 and Isolate_7 displayed a pairwise SNP distance of one (1). These isolates, obtained from urine at Hospital B, also shared similar resistance genes for quinolones, beta-lactams, fosfomycins, and tetracyclines (**Fig 2**).

Phylogenetic analysis of the *E. coli* isolates revealed that the pairwise SNP distances among the isolates ranged from 0 to 47822. Isolate_60, Isolate_40, Isolate_55, Isolate_25, Isolate_36 and Isolate_39 clustered with pairwise SNP distances less than nine (9). These isolates were isolated from wounds at Hospital A and carried similar resistance genes for aminoglycosides, chloramphenicol, beta-lactams, sulphonamides, macrolides, tetracycline and disinfectants. Isolate_29 and Isolate_28 was also recovered from wound from Hospital A and were found to cluster together with a pairwise SNP distance of six (6) (**Fig 3**).

## Discussion

The continuous spread of antibiotic resistant bacteria is a major global public health concern [49]. This study provides granular information on Gram-negatives from clinical sources to inform treatment decisions and surveillance efforts in our settings. Enterobacterales are commonly implicated in clinical infections globally [50] and therefore it was not surprising to find *E. coli* (21/62; 33.87%) and *K. pneumoniae* (13/62; 20.97%) as major pathogens in the collection investigated.

The presence of virulence and antibiotic resistance genes contributes to the ability of these pathogens to colonize various body sites and induce infections [51]. Selective pressure resulting from the widespread use of disinfectants might be a major cause for the observation of *qacE,* a resistant gene for quaternary ammonium compounds frequently occurring among the isolates. Consequently, the use of quaternary ammonium compounds may not only promote tolerance to biocides but also promotes the emergence of multidrug-resistant strains through co-selection. The high prevalence of *qacE* among our isolates underscores the need for integrated antimicrobial stewardship strategies that address both antibiotic prescription and disinfectant usage practices, as well as the implementation of surveillance programs to monitor the dissemination of disinfectant resistance genes in clinical and environmental settings [1,52]. Most of the phenotypically confirmed ESBL-positive isolates (24/30; 80.00%) were found to harbor genes encoding ESBLs, with the majority carrying *bla*_*CTXM-15*._ Moreover, *bla*_*CTX-M-15*_ was found as the major ESBL gene among the isolates which is consistent with findings in Ghana and in other parts of the world [20,53]. Six isolates phenotypically expressed ESBL but contained no ESBL genes. Four of the phenotypically confirmed ESBL-positive isolates did not harbor ESBL genes but possessed AmpC related genes. ESBL expression may be due to various mechanisms and conditions which include over-expression of AmpC genes as suggested in other studies [53,54]. This highlights the complexity of antibiotic resistance mechanisms in bacteria and the need to implement advanced molecular techniques such as WGS in resistance determination. In correlation with our findings, several studies have reported *bla*_*NDM-1*_ producing *K. pneumoniae* [55] but there have been a limited number of research studies that have documented its presence in *P. vermicola*. Carbapenems are last-line antimicrobials [56] therefore it is important to surveil AMR pathogens globally to inform judicious usage of this important agent in management of infections. The detection of plasmid encoded *mcr-10* gene which confers resistance to the last-line drug colistin is a major concern as the gene can be shared among several species [8]. In addtion, the detection of isolates harboring resistance genes for aminoglycosides, fluoroquinolones, tetracyclines, and biocides is of concern, as this will further limit therapeutic options for effective patient management [57].

The finding of several isolates harboring virulence genes supports the characteristic abilities of the pathogens to colonize and cause diseases in their host [58]. The *traT* gene which was found among majority of the isolates has been linked with serum resistance [18,59]. This trait enables organisms to survive and proliferate in the human blood with fully functional complement system and confers the potential to cause serious systemic infections [60].

Detection of IncF plasmid replicons predominantly in *E. coli* (i.e., > 70%) corroborates the findings of other studies conducted in Ghana [19,20]. These plasmids are considered as major transmitters of ESBLs [61,62] and this calls for strengthening of infection prevention and control practices to limit the spread of pathogens with these plasmids in our settings.

*E. coli* ST131 clones have been previously reported in Ghana [19]. These clones are frequent carriers of ESBLs and are responsible for several cases of recurrent urinary tract infections and sepsis hence they are of public health concern [63]. *K. pneumoniae* ST15 and ST147, detected in this study have also been frequently reported in hospital outbreaks and dissemination of antibiotic resistance genes, including *bla*_*NDM-1*_ [64]. ST244 *P. aeruginosa* has been detected elsewhere as a high risk clone due to their multidrug resistance potential [24,65,66].

In this study, *K. pneumoniae* serotype O1 was predominant and this is consistent with past reports that have shown that serotype O1 is predominant in human disease [67]. Multidrug resistant *E. coli* serotype O25:H4 was predominant (6/21; 28.57%) among the *E. coli* collection and were all found to belong to the ST131 clone. This corroborates results from a study conducted in Egypt [68].

The *S. enterica* serova Typhi has been reported globally as a leading cause of mortality and has gained resistance to several antibiotics. Its detection in our collection confirms its spread owing to their acquired genetic features. *S. enterica* serova Lille despite previously reported to cause Salmonellosis outbreaks in humans [69], our work is the first to report its recovery in Ghana from a human source. Surveillance is therefore required due to their high transmissibility. *P. aeruginosa* serotype O6 was the most common *P. aeruginosa* serotype identified and is consistent with findings obtained from other parts of the world [23].

The phylogenetic analysis of *K. pneumoniae* isolates revealed that the isolates: Isolate_58 and Isolate_34 (both originated from Hospital A) had a pairwise SNP distance of four, indicating a close genetic relationship. The resistance gene profiles of the two isolates were also similar which includes resistance to tetracyclines, aminoglycosides, quinolones, beta-lactams, chloramphenicols, sulphonamides, and fosfomycins. Aside the genetic commonality among these two isolates, the diverse sequence types identified from different sources indicate a broad spectrum of *K. pneumoniae* implicated in clinical infections in Ghana [70,71]. A pairwise SNP distance of one was observed between isolates Isolate_15 and Isolate_7 from Hospital B, and both isolates harbor resistance genes conferring resistance to quinolones, beta-lactams, fosfomycins, and tetracyclines.

Pairwise SNP distances of less than nine for isolates: Isolate_60, Isolate_40, Isolate_55, Isolate_25, Isolate_36, and Isolate_39, *E. coli* isolates from Hospital A was observed. These isolates share similar resistance genes, including those conferring resistance to aminoglycosides, chloramphenicol, beta-lactams, sulphonamides, macrolides, tetracycline, and disinfectants. Furthermore, Isolate_29 and Isolate_28 obtained from Hospital A clustered together with a pairwise SNP distance of six. The clustering patterns observed among the isolates suggest a possible spread and transmission of these clones within our settings. Alternatively, these clusters suggest the possible persistence of endemic clones that are stably circulating within the hospital environment rather than recent transmission between patients.

Phylogenetic analyses have shown that *E. coli* strains mainly fall into eight phylogroups (A, B1, B2, C, D, E, F and clade I) with each of them having a unique profile of genes which distinguish its own evolutionary pattern [72]. In the present study, the most common phylogenetic group was B1 (9/21; 42.86%). This group is typically considered to be an environmental lineage, however human extra-intestinal infections such as sepsis has been reported [73]. Group B2 (7/21; 33.33%) contained the second highest number of *E. coli* strains. In general, *E. coli* strains in phylogenetic group B2 carry more virulence factors than strains belonging to phylogroups A, B1, and D, and extraintestinal pathogenic *E. coli* strains belong to this group [74]. A study in a teaching Hospital in Ghana, by Deku *et al.* (2022) reported phylogroup A as the most common group which is not consistent with our findings. The contrast might be due to differences in location or origin of isolates investigated [75].

This study is constrained by the limited number of isolates and the lack of extensive metadata including age, sex, and hospital ward of origin to support statistical and phylogenetic analysis; thus, the findings cannot be broadly generalized. Future studies would benefit from a larger sample size and more comprehensive metadata. Another limitation of this study is the inability to phenotypically test for all antibiotic classes. This approach influences our inability to comprehensively categorize isolates as extensively drug resistant or pan-drug resistant. Furthermore, the absence of long-read sequencing limited the resolution of plasmids.

However, despite these limitations, the study offers insights into the presence of high-risk clones and the carriage of multiple resistance and virulence genes among Gram-negative bacterial species in Ghana, a resource-limited setting.

## Conclusion

Our study offers preliminary insights into the genomic characteristics of Gram-negative bacteria in Ghana. The identification of high-risk clones and the presence of multiple resistance and virulence genes among the isolates underscore the importance of enhancing infection prevention and control measures, antibiotic stewardship programs, and surveillance efforts in this LMIC setting.

## Supporting information

S1 Table The phenotypic and genotypic characterization of the isolates. (XLSX)

S2 Table The genotypic metrics and SNP matrix of the isolates. (XLSX)
